# iRAGu: A Novel Inducible and Reversible Mouse Model for Ubiquitous Recombinase Activity

**DOI:** 10.3389/fimmu.2017.01525

**Published:** 2017-11-10

**Authors:** Marie Bonnet, Leonor Morais Sarmento, Ana C. Martins, Daniel Sobral, Joana Silva, Jocelyne Demengeot

**Affiliations:** ^1^Instituto Gulbenkian de Ciência, Oeiras, Portugal

**Keywords:** recombination activating gene, transgenic mouse model, 4-hydroxytamoxifen induction, estrogen receptor, lymphocyte development, V(D)J recombination

## Abstract

Developing lymphocytes express the recombination activating genes (RAGs) 1 and 2 products that form a site specific recombinase complex (RAG), introducing double strand DNA breaks (DSBs) at recombination signal sequences (RSSs) flanking the V, D, and J gene segments in the antigen receptor loci. The subsequent steps in the reaction consist in the ligation of DSBs by ubiquitous enzymes of the non-homologous end joining DNA repair pathway. This mutagenesis process is responsible for the generation of the very large clonal diversity of T and B lymphocytes, itself allowing the recognition of a virtually open-ended antigenic universe. Sequences resembling RSS are found at high frequency all over the genome, and involved in RAG mediated illegitimate recombination and translocations. Hence, natural and induced ectopic activity of RAG is a threat to the genome only recently underscored. Here, we report and characterize a novel mouse transgenic system for which ubiquitous expression of the recombinase is inducible. In this system, the RAG1 protein is constitutively expressed and functional, while the RAG2 protein, coupled to the estrogen receptor, becomes functionally active upon 4-hydroxytamoxifen (TAM) administration. We describe two transgenic lines. The first one, when introgressed into an endogenous Rag2^−/−^ genetic background is faithfully recapitulating lymphocyte development, repertoire dynamics and cryptic rearrangements, in a TAM-dependent manner. In this model, deprivation of TAM is followed by lymphocyte development arrest, evidencing the reversibility of the system. The second transgenic line is leaky, as the transgenes promote lymphocyte differentiation in absence of TAM treatment. Upon TAM-induction defects in lymphocytes composition and global health reveals the deleterious effect of uncontrolled RAG activity. Overall, this novel transgenic model provides a tool where RAG activity can be specifically manipulated to assess the dynamics of lymphocyte differentiation and the challenges imposed by the recombinase on the vertebrate genome.

## Introduction

B and T lymphocytes are the prerogative of the adaptive immune system thanks to their ability to generate a quasi-infinite diversity of antigen receptors through a mechanism called V(D)J recombination. This process consists in the rearrangement of somatic DNA, to combine one variability (V), possibly a diversity (D), and one joining (J) gene segments together, and comes down to the cutting of the segments by the recombinase complex and the pasting of the broken ends by the ubiquitous non-homologous DNA end-joining repair machinery (NHEJ), within an accessible region called the recombination center [(1)]. Imprecise joining and repair of the gene segments coding ends (CEs) by the NHEJ machinery produce the coding joint (CJ), therefore, adding junctional diversity by removing template nucleotides and adding CG rich *de novo* nucleotide at the junction of the two gene segments (2). On the other side, the two signal ends are ligated together without any editing to form the signal joint (SJ). Both the CJ and the SJ determine the recombinase-dependent signature of the V(D)J recombination mechanism (3).

The recombinase complex is formed by the two lymphoid-specific recombination activating genes (RAG1 and 2) proteins, which are sufficient to induce a double strand DNA break, provided the gene segments are flanked by functional recombination signal sequences [RSSs; (4)], which are the specific target sequences of the recombinase. RSSs are long and considerably degenerated sequences, a feature that may favor a wide range of interactions with RAG and consequently a differential segment usage in the repertoire of antigen receptor genes. In turn, this permissiveness in nucleotide sequences results in an abundant distribution all over the genome of sequences resembling RSS—called cryptic RSS [cRSS; (5, 6)]—which can be occasionally recognized by the recombination machinery and involved in translocation events (7). Actually, illegitimate V(D)J recombination events involving proto-oncogenes and B or T cell receptors (BCR and TCR, respectively) loci are found in most lymphoid leukemias (8, 9). More recently, cRSS and their accessibility to RAG were formally quantified and their purging in vertebrates genomes shown to be limited only to specific gene regions (10, 11). The very tight regulation of RAG expression in developing lymphocytes may well limit unwanted recombination events. In turn, ectopic expression of RAG in non-lymphoid tissues, as occasionally reported in solid tumors [e.g., Ref. (12)], may favor genome alterations.

Both RAG proteins are necessary to the recombination reaction, as shown by the complete absence of rearrangements of antigen receptor genes, and ensuing lack of mature lymphocytes, when either of the two genes is knocked out (13, 14). RAG1 and 2 also drive each step of the recombination reaction by the way they bind DNA, keep broken ends within the recombinase complex and trigger repair of DNA ends by recruiting the NHEJ machinery (15–18). The recombinase complex is tightly regulated at tissue, cellular and molecular levels. First, RAG1 and 2 are both expressed only in lymphocytes, though one or the other are expressed at low levels in different non-lymphoid tissues (19–21). Second, RAG expression is downregulated throughout the G1/S phase of the cell cycle and during the cell divisions following the recombination of an antigen receptor locus (22, 23), which occur primarily after the production of a functional heavy chain of antigen receptor (TCRβ, TCRδ, and IgH loci) and secondly after the expression and the pairing of the light chain to the heavy chain (TCRα, TCRγ, and Igλ or Igκ, respectively) to form the cell surface receptor. And third, the recombinase specifically targets each locus in the appropriate lineage and development stage. For example, this is how the TCRβ locus complete recombination occurs in T cells only, before recombination of the TCRα locus and that a single functional TCRβ is produced per cell, which is called allelic exclusion (24). This last regulation level is driven by changes in chromatin accessibility of the antigen receptor loci that become accessible to the recombinase at the right juncture, to ensure proper lymphocyte development (23).

Recombinase deficiency can be spotted at the level of lymphoid cell differentiation by flow cytometry, by looking at the concomitant expression of cell surface markers to the developmental stages. Thus, V(D)J recombination occurs at the DN3 (CD25^+^CD44^−^CD4^−^CD8^−^) and DP (CD4^+^CD8^+^) stages of T cell development for the TCRβ and TCRα rearrangements, respectively, and RAG-deficient mice exhibit a complete block of thymocytes development at the DN3 stage. Regarding B-cell development, as complete recombination of the IgH is required for the transition to the C′ fraction (B220^+^CD43^+^BP1^+^), RAG-deficient mice show a block of development at the pre-B C fraction (B220^+^CD43^+^BP1^−^), and the absence of IgM^+^ cells (fractions E and F) (25).

To assess the significance of the strict tissue and developmental stage specificities of the *rag* genes expression, we had previously generated transgenic mice in which both *rag* genes were constitutively coexpressed (26) and we reported a premature death of transgenic animals before 4 weeks of age, which was associated with severe lymphopenia.

To overcome this development constrain, we developed a new inducible model for ubiquitous expression of the recombinase. Schatz and coll. had produced an inducible model of RAG activity, which was dependant on tetracycline removal (27). Our system is more straightforward in the sense that RAG1 is constitutively expressed and functional, while RAG2 is coupled to the estrogen receptor (ER), which maintains the RAG2tg sequestered in a cytoplasmic complex and renders the *rag*2tg non functional. 4-Hydroxytamoxifen (TAM) administration uncouples the ER-domain from the RAG2 proteins, which can be then translocated to the nucleus and become functionally active (28). We describe this new RAG-inducible and reversible transgenic model. Using transgenic mice crossed on the RAG2-deficient background, we show that the *rag*1 and *rag*2-ER transgenes are ubiquitously expressed, that their activity rescues the development of B and T lymphocytes upon TAM-administration, and that this induction is reversible. We also show that RAG transgenic activity is functional in non-lymphoid tissues. We describe another line of transgenic mice that is leaky, and which exhibits defects of lymphocyte development.

## Materials and Methods

### Description of the Constructs

H2K-RAG1 and H2K-RAG2ER^TAM^ constructs were previously described (29).

### Mouse Line and Treatment for Induction

Wild-type (WT) and RAG2-deficient (R2^−/−^) used in this study were originally purchased from Jackson Laboratory (Bar Harbor, ME, USA), bred and maintained under specific pathogen-free (SPF) conditions at the IGC animal facility, Portugal. Also, RAG1tg/RAG2tg-ER mice have been established by coinjection of the cut *rag*1tg and *rag*2tg-ER fragments into C57/Bl6 or FVB oocytes. Transgenic founders were identified by PCR (see primers sequences and PCR conditions in Tables S1 and S2 in Supplementary Material). We obtained one line in the C57/Bl6 background that gave progeny (hereafter Tg), which we crossed on a R2^−/−^ background. Additional two lines generated on the Fvb background were then backcrossed on the C57/Bl6 background. The functionality of the RAG2tg-ER was induced by tamoxifen food (CRD TAM400/CreER, Teklad, hereafter TAM) administration, when mice were 5–7 weeks old. For the FACS analyses of the phenotypes, mice were fed 4 weeks with TAM only. For maintained RAG2 activity, transgenic mice were fed 4 weeks with TAM, followed by alternation periods of normal versus TAM food. As for the analysis of the reversibility of the system, mice were either kept under TAM during 1 week and then fed with normal food during 3 weeks, or for 2 weeks on TAM followed by 1 month of normal food.

For bone marrow adoptive transfer, R2^−/−^ recipient mice were sub-lethally irradiated (900 rad) and reconstituted the following day with 5 millions of total bone marrow cells from transgenic or R2^−/−^ mice, injected into the retro-orbital plexus. Normal food and Tam food were given by alternation every week. All mice experimental protocols were in accordance with European, Portuguese and Instituto Gulbenkian de Ciência ethical committee animal guidelines.

### Nucleic Acid Analyses

For transcription analysis, cell suspensions from different organs from WT and Tg R2^−/−^ mice were generated by mechanical disruption of the organs and proteinase K digestion. RNA was performed using RNAeasy kit (Qiagen) as recommended by the manufacturer and cDNA was synthesized as described (30). PCR for the beta-tubulin was performed to assess for the nucleic acid loads, and specific PCR for endogenic and transgenic *rag*1 and *rag*2 were used to determine mRNA levels of each sample. Primer sequences and optimal PCR conditions for each PCR test are shown in Tables S1 and S2 in Supplementary Material, respectively.

We analyzed the Jα usage for the VJα rearrangements as described (31). Briefly, total cDNA was generated from mRNA from total thymocytes from WT and Tg endoR2^−/−^ mice. Semi-nested PCR using degenerated oligos for the Vα segments were used (31) in which EcoRI and XhoI restriction sites were added. PCR fragments were either purified and cloned into EcoRI/XhoI cut pKS vector and sent for sequencing or directly sent for high-throughput sequencing with a MiSeq sequencer, both at the IGC sequencing platform. Reads are available at: https://www.ncbi.nlm.nih.gov/bioproject/PRJNA400571. Original raw paired reads were quality filtered with seqtk (trimfq-q 0.01) and merged with flash. Both merged and unmerged reads were pooled together to pass to the MiTCR software version 1.0.3 (32), using the following command: java-jar-Xmx2G-jar mitcr.jar-pset flex-species mm-gene TCRab_S1S5_filtered_001.all.fastq TCRab_S1S5_filtered_001.all.mitcr.cls, where TCRab_S1S5_filtered_001.all.fastq and TCRab_S1S5_filtered_001.all.mitcr.cls are the input and output files, respectively.

For Bcl11b cryptic recombination analysis, genomic DNA from total thymocytes was prepared by phenol/chloroform extraction and PCR conditions were as described previously (33) to amplify the cryptic rearrangement events. We performed fluctuation PCR by repeating several times (12 or 24) the same amplification with either 60 or 240 ng of DNA for Tg endoR2^−/−^ TAM^+^ and Tg endoR2^+/−^ TAM^+^ mice, respectively (34). PCR fragments were sequenced on the IGC sequencing platform.

### Flow Cytometry

Single-cell suspensions from thymus, lymph nodes, bone marrow, and peripheral blood mononuclear cells (PBMCs) were prepared, counted, and stained as described previously (35). Briefly, 1 × 10^6^ cells per sample were pre-incubated with Fc-block anti-CD16/CD32 followed by staining incubation for 45 min at 4°C with fluorochrome-conjugated antibodies (Antibodies listed in Table S3 in Supplementary Material), before washes. Data were acquired on FACS CyAn™ ADP, using Summit™ software (Beckman Coulter) and analyzed using FlowJo (Tri Star Inc.). Doublets and clumps were excluded from analysis and cell counts and FACS results for live lymphocytes defined as propidium iodide (Fluka) negative cells.

### Enzyme-Linked Immunosorbent Assay (ELISA)

Serum IgM and total IgGs were detected by ELISA. Briefly, Nunc ELISA 96-well plates were coated overnight with 2 µg/mL antimouse IgM (Clone R331.24.12, in house made) or IgG (1030-01). After washings and blocking with gelatin, sera were incubated 1 h at 37°C followed by washings and secondary antibody incubation (1030-05 and 1020-05 for IgG and IgM, respectively). After washings, color was detected using TMB substrate reagent set (555214, BD) as recommended by the manufacturer. 450 nm optical densities were read on Victor3 Multilabel Counter fluorimeter (Wallac PerkinElmer, Turku, Finland), and results analyzed using Igor 3.16 software. Purified mouse IgG (0107-01) and IgM (0101-01) at 2 and 0.5 µg/mL were used as standards. All antibodies were purchased from SouthernBiotech unless otherwise noticed.

### Florescent-Based *In Vitro* Recombination Assay

Primary mouse embryonic fibroblasts (MEFs) derived from day 14 WT and transgenic fetuses were isolated and infected by viral GFPi-mRFP particles (29). To induce RAG2 activity, 16 h postinfection cells were treated with 200 nM of 4-hydroxy-tamoxifen (TAM) dissolved in ethanol (etOH) to a final concentration of 2% etOH per well. Cells were analyzed by flow cytometry on a MoFlo FACS machine (Beckman Coulter).

## Results and Discussion

Here, we present tamoxifen-inducible RAG1tg/RAG2tg-ER mouse models established in our laboratory. We describe the lines that were established on the C57/Bl6 background (hereafter Tg line), and when indicated crossed to an endogenous RAG2-deficient background (hereafter Tg endoR2^−/−^).

### *Rag* Transgenes Are Ubiquitously Expressed *In Vivo*

**Figure 1:**
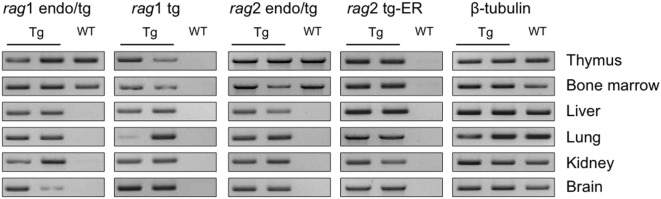
*rag* transgenes are transcribed in lymphoid and non-lymphoid tissues of Tg mice. *rag*1, *rag*2 and β-tubulin transcripts were detected by RT-PCR in thymus, bone marrow, liver, lung, kidney, and brain from one wild-type (WT) and two Tg endoR2^+/+^ mice. Primers endo/tg detect both endogenous and transgenic rag transcripts. Results are representative of three independent experiments analyzing five different mice.

We first confirmed that the *rag* transgenes are expressed ubiquitously in the transgenic mice. Given that the ER-system impedes RAG2 functionality but not expression, there is no need for TAM induction to observe the transcripts of the *rag*2-ER transgene. RAG1 and RAG2 expressions were determined by reverse transcription and PCR. Figure 1 shows the transcription levels of two Tg and one WT animals in the thymus, bone marrow, liver, lung, kidney and brain, for the endogenous and transgenic *rag* transcripts and for the beta-tubulin as a control. Note that the primers used for endogenous *rag* mRNA amplification also amplify the transcript encoded by the transgene (endo/tg, see Table S1 in Supplementary Material for sequences). As expected, *rag* transcripts are detected in thymocytes and bone marrow of both WT and Tg mice, while only the transgenic *rag* transcripts are observed in non-lymphoid tissues, and in all of them, confirming that RAG1tg and RAG2tg-ER are ubiquitously expressed (Figure 1).

### Upon TAM-Induction, *rag2-ER* Transgene Rescue Rag2-Deficiency for Lymphoid Differentiation

*In vivo* functional analysis of the RAG1tg and the RAG2tg-ER was performed by complementation of lymphocyte deficiency of the Rag2-knock out mice (Tg endoR2^−/−^).

**Figure 2:**
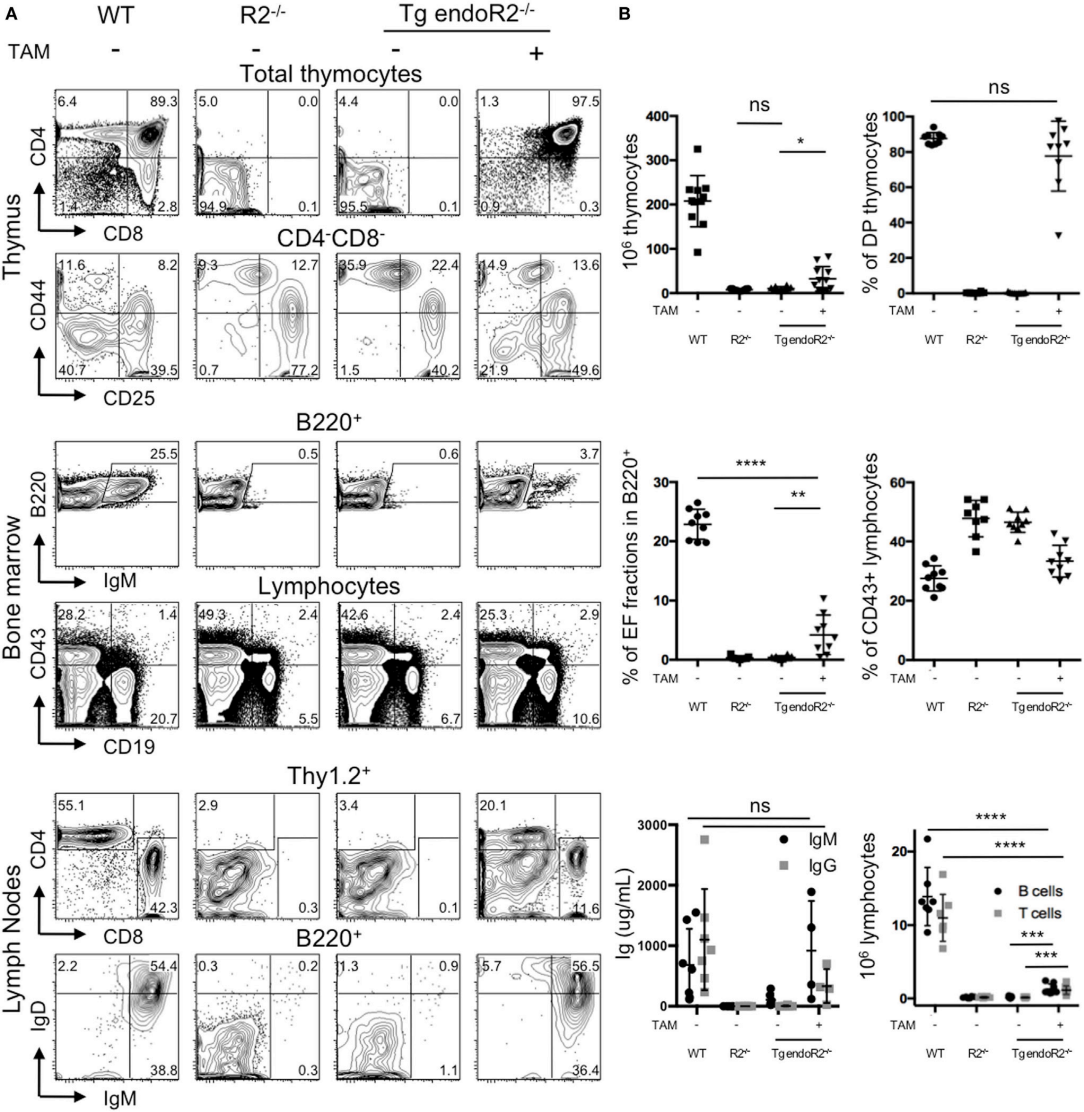
4-Hydroxytamoxifen (TAM) induction of Tg endoR2^−/−^ partially restores lymphocyte development. Young adult Tg-endoR2^−/−^ mice (Tg endoR2^−/−^) were fed with normal (−) or TAM (+) food during 4 weeks before analysis. Untreated wild-type (WT) and recombination activating gene 2 (RAG2)-deficient (R2^−/−^) mice served as control. **(A)** Representative FACS profiles of thymocytes, bone marrow, and lymph nodes cells. **(B)** Full analysis of thymocytes numbers and frequency of thymic DP (upper panels); frequency of E and F fractions and CD43^+^ B cells in the bone marrow (middle panels); concentration of seric Ig and lymphocyte numbers in lymph nodes (bottom panels). *, **, and *** stand for *p*-values <10^−2^, 10^−3^, and 10^−5^, respectively. Results are representative of six independent experiments.

We asked whether the TAM-inducible model was functional *in vivo*. We determined the phenotype of the mice using flow cytometry, by the characterization of the lymphoid compartment in absence of TAM-induction (Figure 2A, third column). First, cell numbers in the thymus from Tg endoR2^−/−^ are very low (in average 3 million cells and in any case below 8 million cells; see Figure 2B), as for RAG-deficient mice. Flow cytometry analysis of the cell surface markers of lymphoid tissues was assessed as previously (26), to describe the differentiation profiles. We analyzed the development of thymocytes and early B cells in the thymus and the bone marrow, respectively, and the mature lymphocytes axillary lymph nodes (Figure 2A). As determined by the CD4/CD8 profiles, the Tg endoR2^−/−^ line exhibits a complete block of development at the CD4^−^CD8^−^ (DN) stage, similar to the R2^−/−^ line. Additionally CD44/CD25 profile in the DN population shows a block at the DN3 stage, as for the R2^−/−^ line. Similarly, there are no mature B cells (fractions E and F Figure 2A middle panels), and a fewer CD19^+^ cells are observed in Tg endoR2^−/−^ line compared to WT, as in the R2^−/−^ background. Consequently, neither mature T nor B cells are detected in the lymph nodes compartment (Figure 2A, bottom lines).

To test the effect of TAM on the lymphoid compartment, WT, R2^−/−^ and Tg endoR2^−/−^ mice were fed with TAM containing food during 4 weeks before analysis. Mice were mildly affected by TAM as indicated by a weight-loss of 5–7%, irrespectively of their genotype (not shown). TAM administration to Tg endoR2^−/−^ mice induces the maturation of thymocytes as attested by the number of thymocytes (Figure 2B) and the presence of DP and SP in the thymus and the peripheral organs (Figure 2A right panels). Also gamma-delta T cells were observed at similar ratios as in WT (Figure S1 in Supplementary Material). Consistent lower numbers of thymocytes in Tg endoR2^−/−^ TAM^+^ animals when compared to WT mice, suggests lower activity of the induced *rag*2 transgene than the endogenous recombinase genes (Figure 2B).

Treatment with TAM also induced mature B cells differentiation in Tg endoR2^−/−^ mice, found in the bone marrow (as attested by the presence of fraction E/F cells, i.e., IgM^+^ cells) and in the lymph node (with IgM^+^ and IgD^+^ cells), and frequencies of CD19^+^ cells were partially restored (Figure 2A middle panels). Nevertheless, the proportion of IgM^+^ cells was much lower in the bone marrow of Tg endoR2^−/−^ TAM^+^ than in WT animals (3.7 versus 25.5%; see Figure 2A). We asked if there would be an impact on circulating antibodies levels in the sera of these animals, and we measured circulating Ig levels by ELISA. Figure 2B bottom left plot shows that treatment of Tg endoR2^−/−^ by TAM overall rescued IgM and IgG production, with a large distribution of Ig levels, but not significantly different when compared to WT animals (*p*-value > 0.1). We conclude that induction of the *rag*2 transgene by tamoxifen rescues the production of circulating antibodies in the sera of RAG2-deficient mice.

Altogether, these results show that the RAG1tg/RAG2tg-ER mouse model is inducible *in vivo* and successfully complement a null mutation for B and T cell development.

### Persistence of RAG Activity in the Thymus

**Figure 3:**
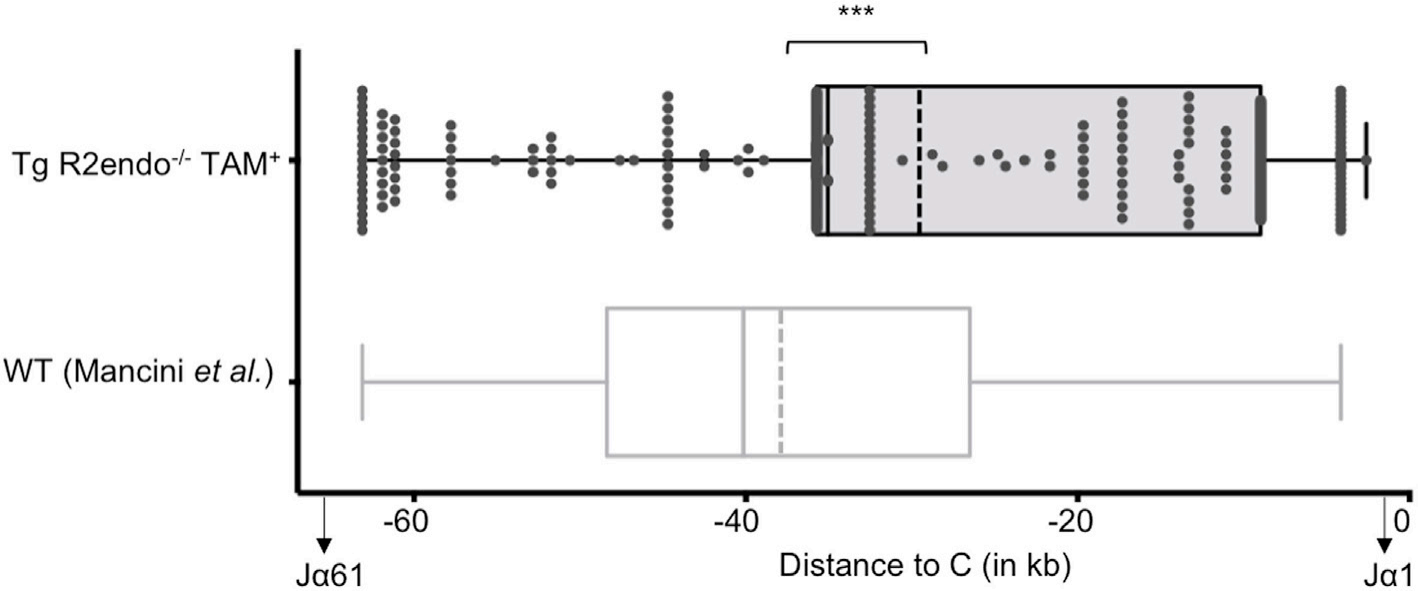
The T cell receptor (TCR) Jα repertoires of 4-hydroxytamoxifen (TAM)-induced Tg endoR2^−/−^ and wild-type (WT) mice are significantly different. Tg-endoR2^−/−^ mice (TgendoR2^−/−^) were fed with normal TAM (+) food during 4 weeks before analysis. PCR and HTS sequencing of TCRα rearrangements were performed and represented as follow: each dot is a rearrangement, and the *x*-axis represent the distance between the Jα used in the rearrangement and the constant (C) region. The doted vertical bars represent the average distances to (C) of the Jα used in rearrangement in TAM-induced Tg endoR2^−/−^ (upper boxplot) and WT mice (bottom boxplot, from Mancini et al.). *** stands for a *p*-value <10^−4^.

We then asked if RAG activity is persistent in transgenic animals, and analyzed the Jα repertoire of the TCRα locus, which undergoes successive secondary rearrangements during the DP stage of thymocytes development (36). This particularity results in a bias of Jα usage in favor of the 5′ available gene segments when extra cycles of secondary rearrangements are allowed. We determined the Jα usage frequency of pooled cDNA from three Tg endoR2^−/−^ fed with TAM food during 4 weeks, and determined the frequency of Jα gene segments usage as described in Mancini et al. (31), and using HTS (see Materials and Methods). We compared the repertoire obtained for the Tg endoR2^−/−^ mice with the Jα repertoire of WT described in Mancini et al. and observed a significant bias for the Jα segments that are more proximal to the constant region in transgenic mice (Figure 3, *p* < 0.001). Of note, we also monitored Jα usage in WT mice by individual cloning (22 rearrangements sequenced), and observed a similar repertoire as described in Mancini et al. (*p* = 0.28, data not shown). Analysis of the sequences of TCRα rearrangements from Tg endoR2^−/−^ from TAM-induced mice showed a RAG/NHEJ-mediated recombination signature, with deletion of CE nucleotides, addition of *de novo* nucleotides rich in C/G bases and with potential palindromic nucleotides, as shown on Table 1. These results indicate that RAG2tg activity is persistent after the DP stage and enable efficient secondary recombination of the TCRα locus.

**Table 1:** Sequences of six TCRα coding joints exhibit the features of the RAG-mediated signature.

Rearrangement	5′ sequence	N/P	3′ sequence
TRAV13-TRAJ39	ACTCAGGCACTTATCTCTGTGCTATGG – –	**CC**C**C**	–GAATAATAATGCAGGTGCCAAGCTCACATT
TRAV4-TRAJ56	ACTCAGGCACTTACTTCTGTGC – – – –	TGCTGCGC	– – – CTGGAGGCAATAATAAGCTGACTTT
TRAV4-TRAJ56	ACTCAGGCACTTACTTCTGTGCTG – – –	**C**TGCGC	– – – CTGGAGGCAATAATAAGCTGACTTT
TRAV4N-4-TRAJ58	GCGACTCAGCCAAGTACTTCTGTGC – – –	CCCCCCGGG	– – – –AGGCACTGGGT_TAAGCTGTCATT
TRAV17-TRAJ58	CTCAGCCAAGTACTTCTGTGCACTGGAGGG	**CC**GAG	– – – AAGGCACTGGGTCTAAGCTGTCATT
TRAV17-TRAJ58	CTCAGCCAAGTACTTCTGTGC – – – – –	TGCCCCCCCGGG	– – – –AGGCACTGGGTCTAAGCTGTCATT

### Transgenic RAG Activity Promotes Also Illegitimate Rearrangements

**Figure 4:**
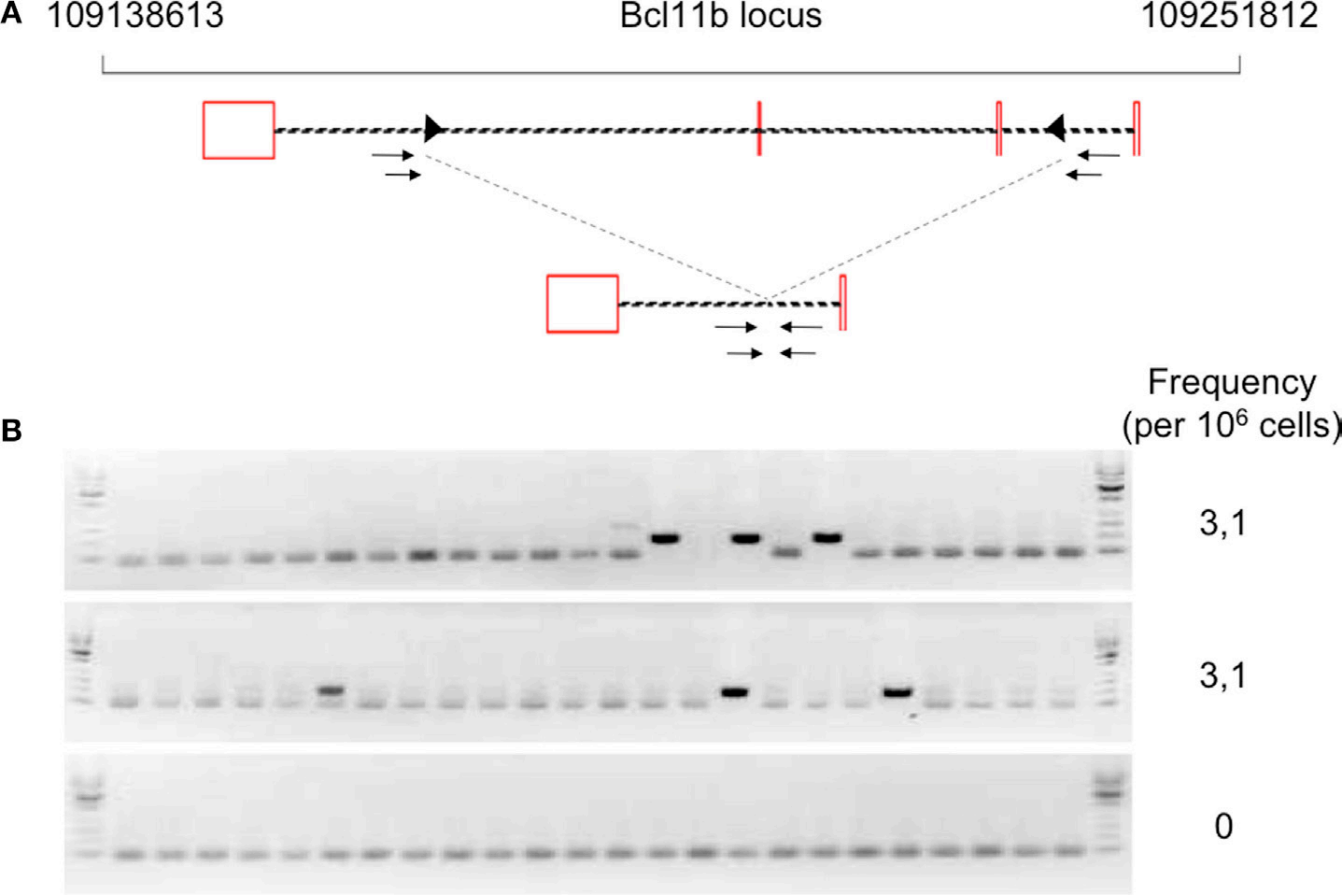
Occurrence of Bcl11b cryptic recombination in 4-hydroxytamoxifen (TAM)-induced Tg endoR2^−/−^ animals. **(A)** Bcl11b locus, cryptic recombination sites and nested primers used in the fluctuation PCR analysis. Bcl11b exons are represented by red boxes, with introns represented as dashed lines connecting these boxes (note that bcl11b is on the reverse strand); cRSS are depicted as triangles within the introns, and primers used for the nested PCR are depicted by arrows. The bottom line shows an illegitimate Bcl11b recombination. **(B)** Fluctuation PCR analysis for each mouse concerned 24 reactions, each with 240 ng of genomic DNA, equivalent to a total of 0.96 million cells. Each row is the analysis of the total thymocytes of one Tg endoR2^−/−^ TAM^+^ mice. Recombination was detected in two out of three mice, at the frequency indicated in the right column.

The development of B and T cells is only partially restored in Tg endoR2^−/−^ animals induced for 4 weeks with TAM (see Figure 2). We next asked whether RAG activity from transgenic origin was sufficient to induce illegitimate recombination, and tested the described Bcl11b cryptic rearrangement to this end (see Figure 4A). We amplified the cryptic rearrangements in WT animals and in three Tg mice induced by TAM for 4 weeks. We used fluctuation PCR of total thymocytes DNA to assess frequencies of Bcl11b recombination which was similar in WT (25 rearrangements per million thymocytes) and RAG-competent Tg animals (27.7 rearrangements per million thymocytes), and in the range of frequencies documented previously [23–50 rearrangements per million thymocytes in Ref. (37) and Ref. (33), respectively; data not shown].

We then observed that Bcl11b cryptic rearrangements were also detectable in two Tg endoR2^−/−^ animals induced by TAM, albeit approximately 10 times less frequently than in WT [(33, 37); Figure 4B; Table 2]. Such cryptic rearrangements were not detected in the 1 million cells equivalent DNA tested for the third Tg endoR2^−/−^ TAM^+^ animal (Figure 4). These results indicate that cryptic recombination does occur in transgenic animals, and therefore that 4 weeks of transgenic RAG activity is sufficient to induce illegitimate recombination.

**Table 2:** Coding joints sequences of Bcl11b cryptic recombination events exhibit the features of the RAG-mediated signature.

5′ sequence	N/P	3′ sequence
GGGCACTAACAAGGAAAGAAATTAATCTCA	**T**T	– – TGTCTGTGTGTCTGTGTGTCCCTTCTA
GGGCACTAACAAGGAAAGAAATTAATCTCA	GA	TGTGTGTCTGTGTGTCTGTGTGTCCCTTCTA
GGGCACTAACAAGGAAAGAAATTAATCTCA	**T**T	– – – – – – TGTCTGTGTGTCCCTTCTA
GGGCACTAACAAGGGAAGAAATTAATCT –	-	– – – TCTGTGTGTCTGTGTGTCCCTTCTA
GGGCACTAACAAGGAAAGAAATTAATC – –	**C**	– -GTGTCTGTGTGTCTGTGTGTCCCTTCTA
GGGCACTAACAAGGAAAGAAATTAA – – –	ACCGTT	– – TGTCTGTGTGTCTGTGTGTCCCTTCTA
GGGCACTAACAAGGAAAGAAATTA – – –	**T**CCACGGTT	– – TGTCTGTGTGTCTGTGTGTCCCTTCTA
GGGCACTAACAAGGAAAGAAATT – – – –	CCCTGAG	– – –GTCTGTGTGTCTGTGTGTCCCTTCTA
GGGCACTAACAAGGAAAGAAATT – – – –	-	– – – – – – –GTCTGTGTGTCCCTTCTA
GGGCACTAACAAGGAAAGAAATTA – – –	**T**CT**C**	– – – – – – –GTCTGTGTGTCCCTTCTA
GGGCACTAACAAGGAAAGAAATTAATCTC–	TTCA**A**	TGTGTGTCTGTGTGTCTTGGTGTCCCTTCTA
GGGCACTAACAAGGAAAGAAA – – – – –	CCC	– – TGTCTGTGTGTCTGTGTGTCCCTTCTA
GGGCACTAACAAGGAAAGAAATTAATCTCA	G**A**	TGTGTGTCTGTGTGTCTGTGTGTCCCTTCTA
GGGCACTAACAAGGAAAGAAATTAATC – –		–GTGTGTCTGTGTGTCTGTGTGTCCCTTCTA
GGGCACTAACAAGGAAAGAAATTAATCT –	-	– – – TCTGTGTGTCTGTGTGTCCCTTCTA

### Upon TAM-Weaning, Transgenic RAG Activity Ceases

**Figure 5:**
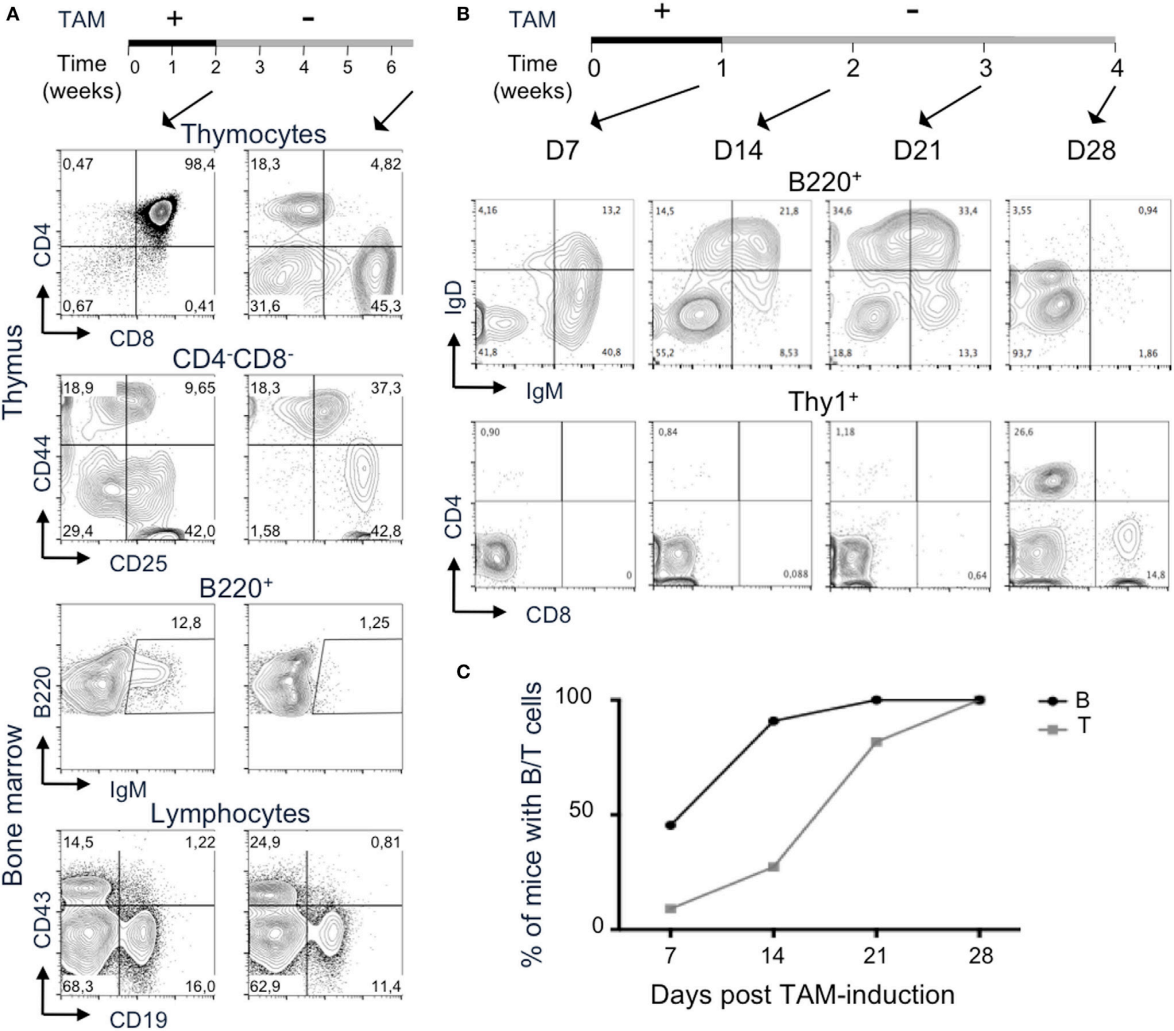
Termination of 4-hydroxytamoxifen (TAM) administration prevents further lymphocyte development. **(A,B)** The upper lines represent the timelines of normal and TAM food administration in Tg endoR2^−/−^ animals, arrows indicate the time at analysis. **(A)** Administration of TAM during 2 weeks is sufficient to induce mature lymphocytes differentiation (left panels), while withdrawal of TAM for 4 weeks arrests lymphoid development. Representative FACS profiles of developing T cells in the thymus (upper panels) and B cells in the bone marrow (bottom panels). Data are representative of 3 independent experiments. **(B)** B cell development precedes T cells differentiation. Representative FACS profiles of B220^+^ peripheral blood mononuclear cell (PBMC, upper panels) and Thy1^+^ (lower panels) cells, after 1 week of TAM administration (D7) or followed by 1, 2, or 3 weeks of normal food. IgM^+^ and IgD^+^ B cells are observed from the first week of analysis, while only a few CD4^+^ cells are detected at that stage. Clear CD4^+^ and CD8^+^ T cells populations are detected at day 28. Data are representative of three independent experiments. **(C)** B cells consistently appear before T cells in peripheral blood. Kinetic analysis of B and T cells frequency in blood of Tg endoR2^−/−^ animals treated along the **(B)** regimen (*N* = 11 mice).

We then tested whether the induction of RAG2tg-ER was reversible. We administrated TAM to Tg endoR2^−/−^ mice during 2 weeks, after what we switched for normal food. As a control, FACS profiles of thymus and bone marrow analyses from a Tg endoR2^−/−^ mouse right after 2 weeks of TAM administration (see Figure 5, left panels) show mostly DP thymocytes, though immature single positives cells start appearing (around 1%). The presence of mature fractions E to F cells attests for B cell development. These results show that a period of 2 weeks of TAM is sufficient to induce B and T cell differentiation beyond the blockade induced by RAG deficiency.

Tamoxifen elimination half-life is about 5–7 days in human, but may be up to 21 days (38). We carried the TAM-deprivation period (TAM-OFF) during 1 month and mice were sacrificed at the termination point of the experiment (6.5 weeks postinduction, right panels in Figure 5A).

The CD4/8 profile of Tg endoR2^−/−^ animals revealed no or very few thymocytes and also showed persistent SP cells (Figure 5). The FACS profiles also revealed a block of development at the DN3 stage, as seen for the R2^−/−^ line (Figures 1A and 5, CD44/25 profile). Likewise, bone marrow cells exhibit few IgM^+^ cells and show a reduced frequency of CD19^+^ cells, although the block at the CD43^+^CD19^+^ is not marked (Figure 5). Similar results were observed for the two other mice of the same experiment and for three animals induced 1 week with TAM followed by 3 weeks of normal food before sacrifice and analysis of lymphoid organs (data not shown and Figure 5B).

All in all, this analysis reveals that the functionality of the RAG2 proteins from transgenic origin can be interrupted by weaning off tamoxifen, and that the inducible system we developed is also reversible.

### The Inducible RAG-Activity System As a Model for the Analysis of T and B Cell Development Kinetics

During the experiment in which Tg endoR2^−/−^ mice were fed on TAM during 1 week followed by 3 weeks of normal food (Figure 5B), a sample of peripheral blood was analyzed every week for the presence of B and T cells. PBMC analysis showed that 1 week of TAM induction was sufficient to observe mature lymphoid cells in the peripheral blood (Figure 5C). This analysis also revealed that B cells appeared earlier than T cells (about 2 weeks earlier), and that among T cells, CD4^+^ appeared before CD8^+^ single positive T cells, a result consistent in 4 independent experiments analyzing 11 mice. The development of thymocytes from DN1 to DP (after TCRα recombination) takes about 7 or 8 days (39), a time scale similar to that required for peripheral B cell to be detectable in our system. It is also well established that migration through thymic epithelial cortex and medulla associates with step wise selection events purging nascent T cells in a much more severe manner than what is measurable for the B lineage in the bone marrow. Absence of DP in the thymus from Tg endoR2^−/−^ mice fed for 1 week with TAM food (data not shown) indicates that the delay is anterior to migration to the periphery, and in turn suggests that rearrangements are more efficient at the Ig than at the TCRαβ loci.

Finally, upon TAM weaning, B cells vanished earlier than T cells (see day 28 in Figure 5B), in agreement with the poorer lymphopenia-induced proliferative capacity of mature B than T cells. We observed some discrepancies between individuals as to the timing of mature cells appearance and the amount of B and T cells in the PBMC, which might be due to differences in TAM intake between mice. However, we could not correlate low number of mature cells in the periphery with high weight loss (i.e., with tamoxifen-intake).

### Transgenic RAG Activity Can Be Maintained

**Figure 6:**
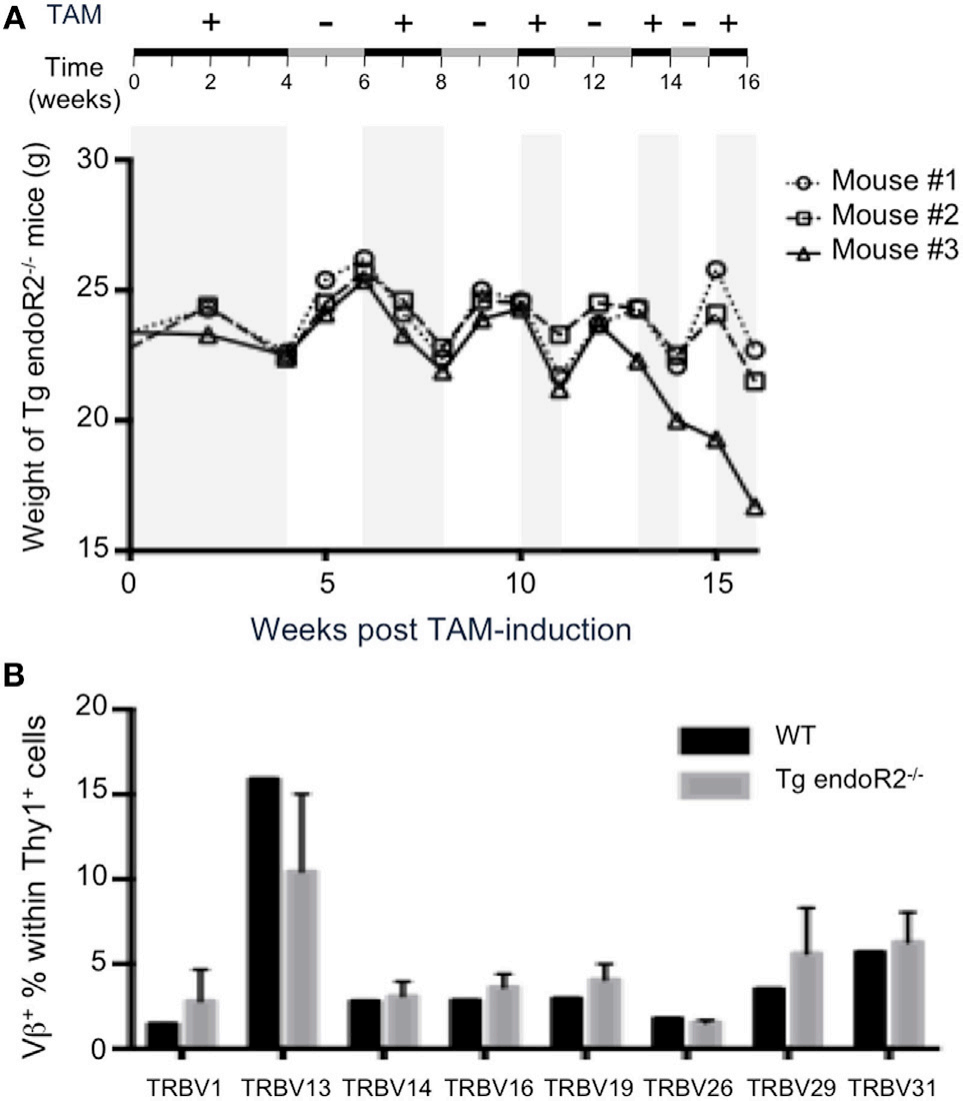
Alternated on and off 4-hydroxytamoxifen (TAM) administration reduces toxicity and promotes completion of the Vbeta repertoire. **(A)** Tg endoR2^−/−^ mice were treated for 16 weeks with alternate periods on (+) and off (−) TAM food (upper scheme), and body weight kinetics established along the treatments. **(B)** By week 16 of the alternate treatment in **(A)**, thymocytes were analyzed by FACS, and wild-type (WT) thymocytes served as control. Shown is the proportion of Thy1^+^ thymocytes expressing each of the 8Vβ tested.

As mice loose weight under TAM treatment (up to 10% of total weight per week), we optimized the system to improve animal welfare while maintaining RAG2tg activity. Therefore, we let mice feed on TAM during 4 weeks and then alternated between TAM and normal food (see Figure 6A upper line). The weight of animals was systematically increasing and decreasing during normal versus TAM periods, respectively (Figure 6A). The presence of B and T cells in peripheral blood was observed through the full duration of the experiment until week 16 (data not shown), and thymus and bone marrow from the animals at the termination point exhibit similar profiles as described in Figure 2, with mature B and T cells in the bone marrow and thymus, respectively, as well as in the lymph nodes (Figure S2 in Supplementary Material), showing that alternation between normal and TAM food is enough to maintain RAG-activity. Additionally, the Vβ repertoire of these three transgenic mice was normal, since the expression of the 8Vβ in Thy1^+^ thymocytes from the three Tg endoR2^−/−^ mice was similar to the WT repertoire (Figure 6B). Of note, the repertoire in WT animals was strikingly similar to that observed previously (30, 40).

### A Model of RAG Overexpression

In addition to the transgenic model described above, which was generated directly on a C57/Bl6 background, two transgenic lines initially generated on the Fvb background were backcrossed to the C57/Bl6 background. One of these presented a phenotype similar to those described above (data not shown), while the other differed for several features. Here, we describe the latter transgenic line, which we refer to as the Tg^hi^ line.

**Figure 7:**
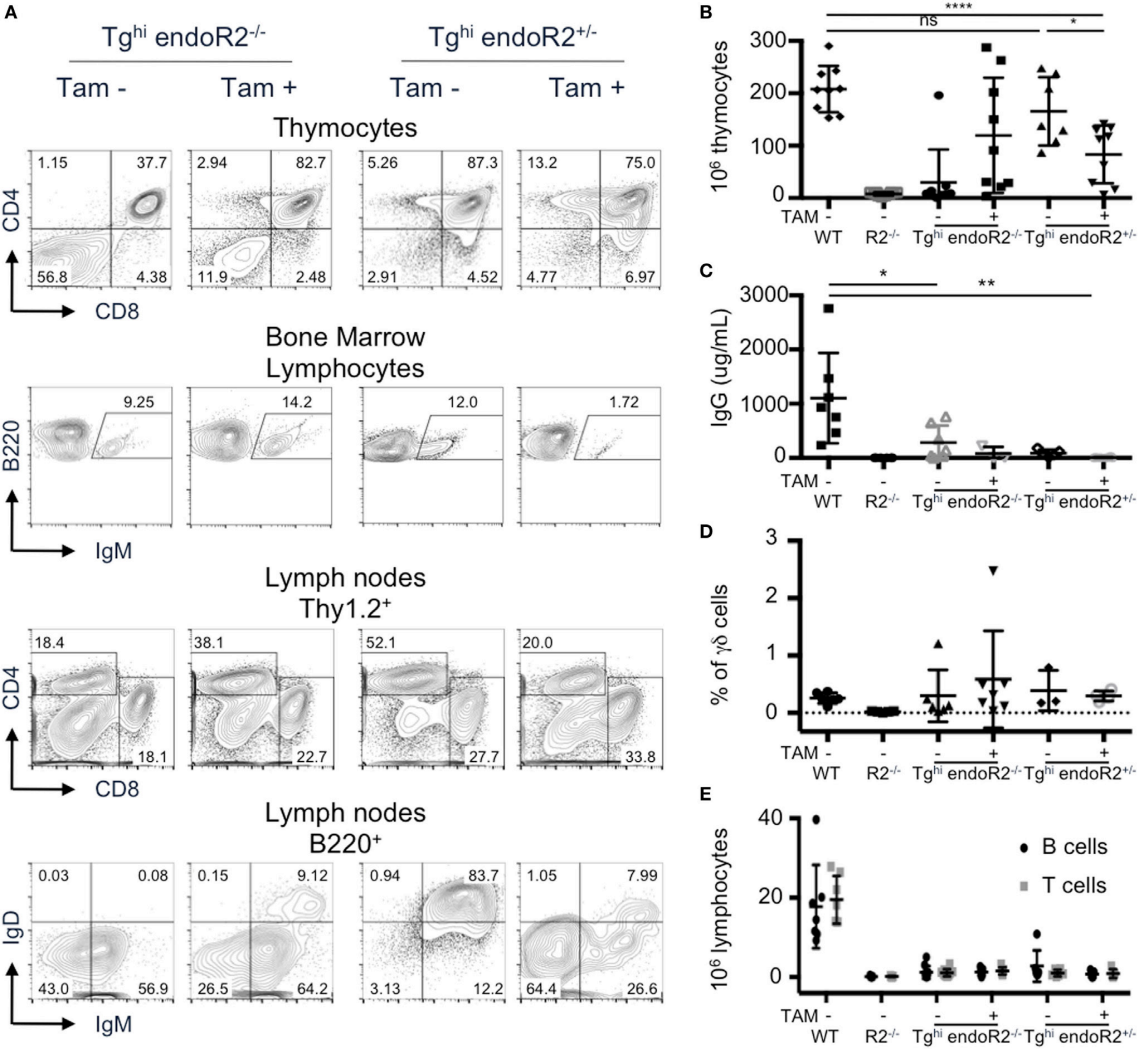
The Tg^hi^ line is leaky and exhibits impaired lymphocyte development/maintenance. Tg^hi^ endoR2^−/−^ and Tg^hi^ endoR2^+/−^ mice were induced (TAM+) or not (TAM−) for 4 weeks before analysis. Wild-type (WT) and Rag2^−/−^ mice served as control **(A)** Representative FACS analysis of thymocytes, bone marrow, and lymph nodes in Tg^hi^ mice. **(B)** Total thymocytes numbers. Tg^hi^ endoR2^−/−^ TAM-mice have higher number of thymocytes than R2^−/−^ mice, and Tg^hi^ endoR2^+/−^ mice exhibit a significant decrease of thymocytes when treated by TAM (*p = 0.016 and ****p < 0.0001 compared to untreated Tg^hi^ endoR2^+/−^ and WT mice, respectively). **(C)** Serum IgG concentration was assessed by quantitative enzyme-linked immunosorbent assay (ELISA). Tg^hi^ mice, whether endoR2^−/−^ or ^+/−^, present significantly lower seric IgG levels than WT animals (*p = 0.03 untreated Tg^hi^ endoR2^−/−^, and **p = 0.008 TAM^+^ Tg^hi^ endoR2^+/−^ mice, respectively). **(D)** Frequency of γδ T-cells in thymocytes was determined by FACS analysis. No statistical differences were found between the different groups. **(E)** Absolute number of mature T and B cells in pooled lymph nodes reveals severe lymphopenia in Tg^hi^ mice, irrespective of functional endoRag2 or TAM treatment. Results are representative of six independent experiments.

We analyzed thymus and bone marrow cells of Tg^hi^ endoR2^−/−^ mice maintained under normal food. Unexpectedly, DP and SP thymocytes, as well as IgM^+^ B cells (Figure 7A left panels) were readily detectable. Consistently with these results, Tg^hi^ endoR2^−/−^ mice present bigger thymi than R2^−/−^ mice, which was confirmed by thymocyte count (Figure 7B). Furthermore, seric IgG and IgM were detectable in Tg^hi^ endoR2^−/−^ mice (Figure 7C; Figure S3A in Supplementary Material). Genotyping data suggested higher copy number, and RT-PCR analysis supported more abundant tg-rag1 and tg-rag2ER transcripts in this Tg^hi^ line when compared to the two others transgenic lines produced (not shown). We conclude that the Tg^hi^ line presents leakiness, likely related to the inefficient cytoplasmic retention of overexpressed RAG2-ER protein, and as reported previously for specific ERT fusion transgenes [e.g., Ref. (41)].

Treatment with TAM food, further enhanced B and T cell development in Tg^hi^ endoR2^−/−^ mice, as indicated by increased frequency of mature T and B cells and increased thymocytes numbers, reaching for some mice values similar to those observed in WT mice (second column of panels on Figures 7A,B). The high variability in thymocytes number was not age-related and may be a consequence of variable TAM intake, or else related to tgRAG toxicity, as evidenced below.

We also bred the Tg^hi^ line on the RAG2-competent background. We observed similar FACS profiles, as well as similar number of thymocytes between these mice and WT animals (Figure 7A third column and Figure 7B). Interestingly, TAM induction of Tg^hi^ (RAG-competent) mice resulted in a significant reduction of thymocytes numbers compared to WT and non-induced Tg^hi^ mice (*p* < 0.0001 and *p* = 0.016, respectively), a feature not observed for the RAG-competent Tg line described in Sections “Description of the Constructs” to “Transgenic RAG Activity Can Be Maintained” (Figure S3C in Supplementary Material). This reduction was associated with a lower frequency of DP cells (Figures 7A,B and D; Figure S3B in Supplementary Material) and quasi absence of T cells in the periphery (Figure 7E). The population of B220^+^ IgM^+^ (fractions E and F) in the bone marrow was also significantly reduced (*p* = 0.01, Figure S3D in Supplementary Material). Finally, the levels of total IgM and IgG in the sera of TAM-induced Tg^hi^ mice were very low and similar to those observed in RAG2-deficient animals (Figure S3A in Supplementary Material; Figure 7C). These results strongly suggest a deleterious effect of RAG over-expression (i.e., endogenous plus transgenic) on B and T cell development, as we had discussed in our previously reported model of constitutive RAG1 and RAG2 overexpression (26).

**Figure 8:**
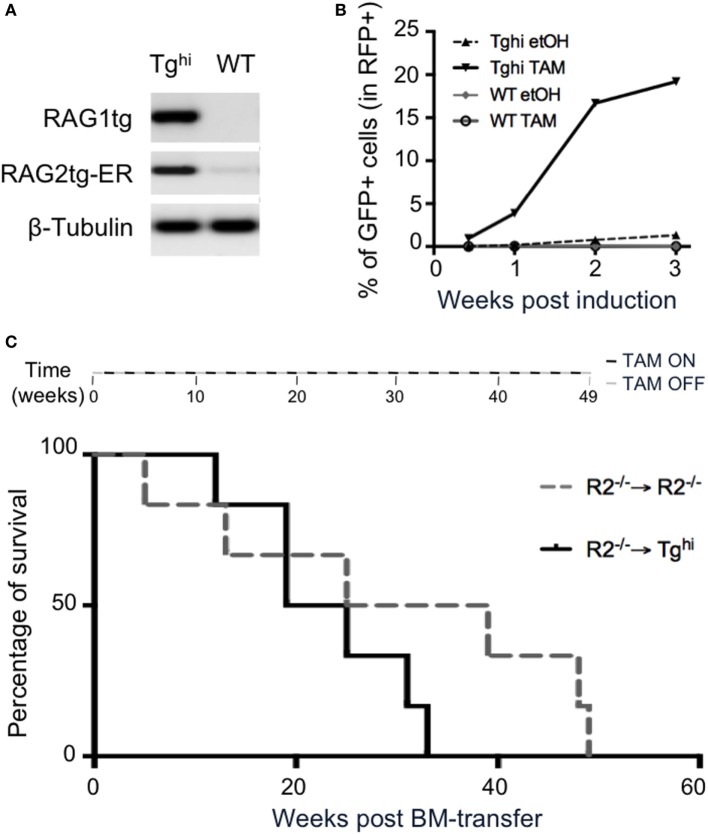
4-Hydroxytamoxifen (TAM)-induction of recombination activating gene (RAG) activity in non-lymphoid organs. **(A,B)** Mouse embryonic fibroblasts (MEF) cultures were established from Tg^hi^ and wild-type (WT) fetuses. **(A)** RT-PCR analysis revealed readily detectable Tg *rag*1and Tg *rag*2-ER transcripts in Tg^hi^ MEF. **(B)** RAG activity was assessed using the GFPi recombination assay. Upon infection with viral GFPi-mRFP particles, transgenic MEF were treated with either 2% ethanol (etOH) or 2% ethanol containing 200 nM of 4-hydroxy-tamoxifen (TAM) during 3 weeks. GFP expression revealed by FACS analysis accounts for induced RAG activity. Results are representative of two independent experiments. **(C)** The upper scheme represents alternated on and off TAM administration during the 49 weeks duration of the experiment. The graph shows survival of R2^−/−^ (gray dotted line) and Tg^hi^ (black line) mice reconstituted with R2^−/−^ bone marrow. Life span was not significantly different between the groups. *N* = 6 for each group.

We next asked if the transgenic recombinase was functional in non-lymphoid cells. We used 14 days MEF from Tg^hi^ animals and confirmed they express the transgenes (Figure 8A). For functional assay, we took advantage of the GFPi reporter of RAG activity we developed earlier (29). This assay allows to quantify RAG activity through the expression of GFP, itself conditioned by an event of RAG mediated inversion. The construct also contained mRFP, which serves as a marker of efficient gene transfer. WT and Tg^hi^ MEF were infected by GFPi viral particles and treated during 3 weeks either with ethanol (etOH), as a negative control, or with TAM. FACS analysis revealed the presence of GFP^+^ cells in transgenic MEF under TAM induction, with levels of GFP expression increasing with time of induction (Figure 8B; Figure S4A in Supplementary Material). Non-induced Tg^hi^ MEF exhibit low levels of GFP expression, confirming the leakiness of the transgene in this mouse line. Of note, infected MEF from the non-leaky Tg line exhibit up to 5% of GFP expression after 3 weeks of induction (versus 20% for the Tg^hi^ MEF, Figure 8B; Figure S4B in Supplementary Material), while no GFP expression was observed without TAM-induction. Altogether, these data confirm that *rag*1 and *rag*2-ER are functional transgenes in a non-lymphoid tissue, and that this activity is TAM-inducible.

We next questioned whether ectopic RAG activity is *per se* deleterious. To limit the transgene expression to non-lymphoid tissues, we reconstituted Tg^hi^R2^−/−^ mice with R2^−/−^ bone marrow cells and provided TAM every other week, for the whole duration of the experiment (see Materials and Methods). Control animals were R2^−/−^ hosts that received R2^−/−^ bone marrow. A survival analysis revealed 50% death at the same time in both experimental and control animals (Figure 8C). In later time points control mice appeared to survive longer, a result that was not statistically significant. This result is to be compared with those we reported earlier revealing that mice engineered to express RAG ubiquitously and constitutively present severe lymphopenia and die before 4 weeks of age (26). By lack of evidence that immunological disorder could lead to early death in SPF mice, we postulated a non-immunologic cause of death. It is today well established that several primary immunodeficiencies can cause very early lethality, and together with our present results it is plausible the main cause of very early death in this previous work was immunological. Nevertheless, ectopic expression of Rag early in ontogeny may have deleterious effect not revealed in adults.

Others already reported that non-physiological or persistent RAG activity is associated with tumorigenesis and genomic instability in non-lymphoid tissues (42, 43). We also crossed Tg^hi^ mice with p53-deficient (p53^−/−^) animals, reasoning this system may allow investigating further the contribution of RAG activity to lymphoid and non-lymphoid tumors. However, control experiments revealed a dramatic decrease in viability of control p53^−/−^ mice fed TAM, invalidating the approach.

In conclusion, the sum of our analysis validates our tamoxifen-inducible and reversible mouse model of ubiquitous RAG activity (iRAGu). The versatility of this system, by allowing the phasing of RAG activity, should serve to dissect further the molecular and cellular dynamics of lymphocyte development. The model also allows ectopic RAG activity and this feature should serve to further investigate the challenges imposed by the recombinase on the vertebrates genome.

## Ethics Statement

This study was carried out in accordance with the recommendations of the animal guidelines from the Instituto Gulbenkian de Ciência ethical committee, in accordance with European and Portuguese animal guidelines. The protocol was approved by the Instituto Gulbenkian de Ciência ethical committee.

## Author Contributions

MB, LS, and JD designed the experiments and analyzed the results. MB, LS, AM, and JS performed the biological experiments. MB and DS analyzed HTS data. MB and JD wrote the manuscript.

## Conflict of Interest Statement

The authors declare that the research was conducted in the absence of any commercial or financial relationships that could be construed as a potential conflict of interest.
