# Supplementary material for: iRAGu: A Novel Inducible and Reversible Mouse Model for Ubiquitous Recombinase Activity
Source: Front Immunol. 2017 Nov 10;8:1525. doi: 10.3389/fimmu.2017.01525 (PMC5686385; doi:10.3389/fimmu.2017.01525)
Supplement: Supplementary file 1 [file Presentation_1.PDF]

Supplementary Figure 1

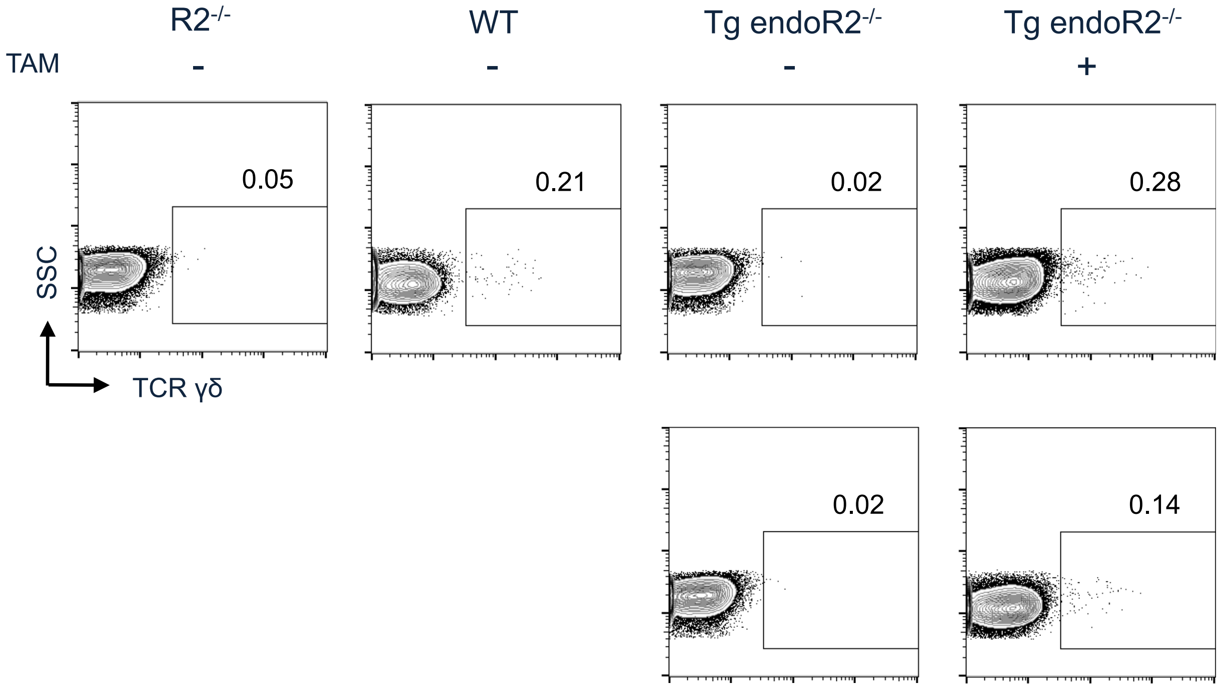

Supplementary Figure 1. **γδT-cells proportions are restored in induced Tg endoR2$^{-/-}$ mice.** Proportions of γδT cells in thymi of RAG2-deficient (R2$^{-/-}$), wild type (WT) and transgenic Rag2-incompetent (Tg endoR2$^{-/-}$) mice fed with normal (-) versus TAM food during 4 weeks (+) (2 animals each). γδT-cells are absent in Tg endoR2$^{-/-}$ animals. The induction of the *rag2-ER* transgene by tamoxifen restores the levels of γδT-cells observed in WT. Data is representative of 3 independent experiments.

Supplementary Figure 2

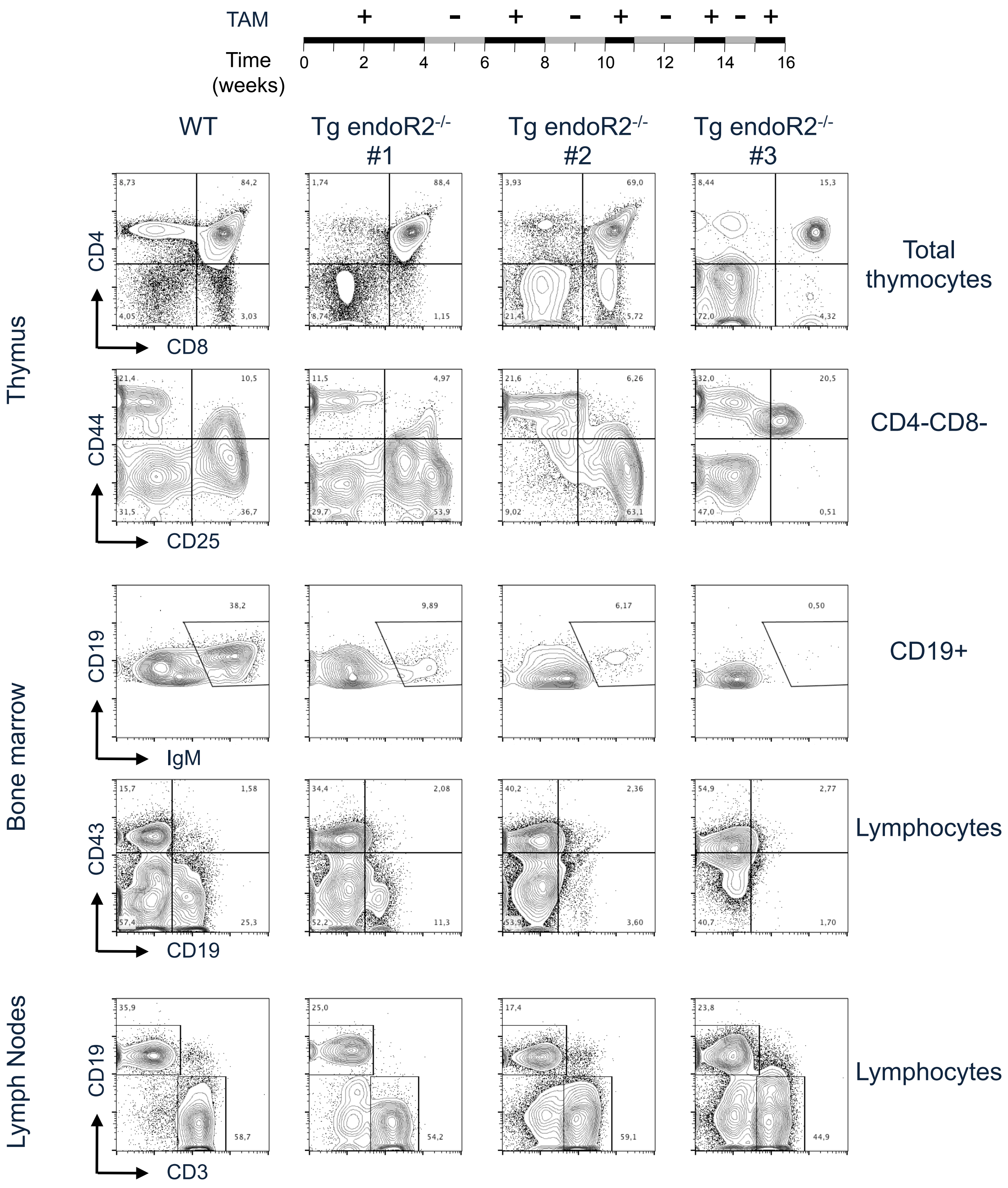

Supplementary Figure 2. **Lymphocyte development does not depend on TAM intake duration.** FACS profiles show similar profiles after 16 weeks of normal and TAM food alternations as after only 4 weeks of TAM administration (see figure 2).

Supplementary Figure 3

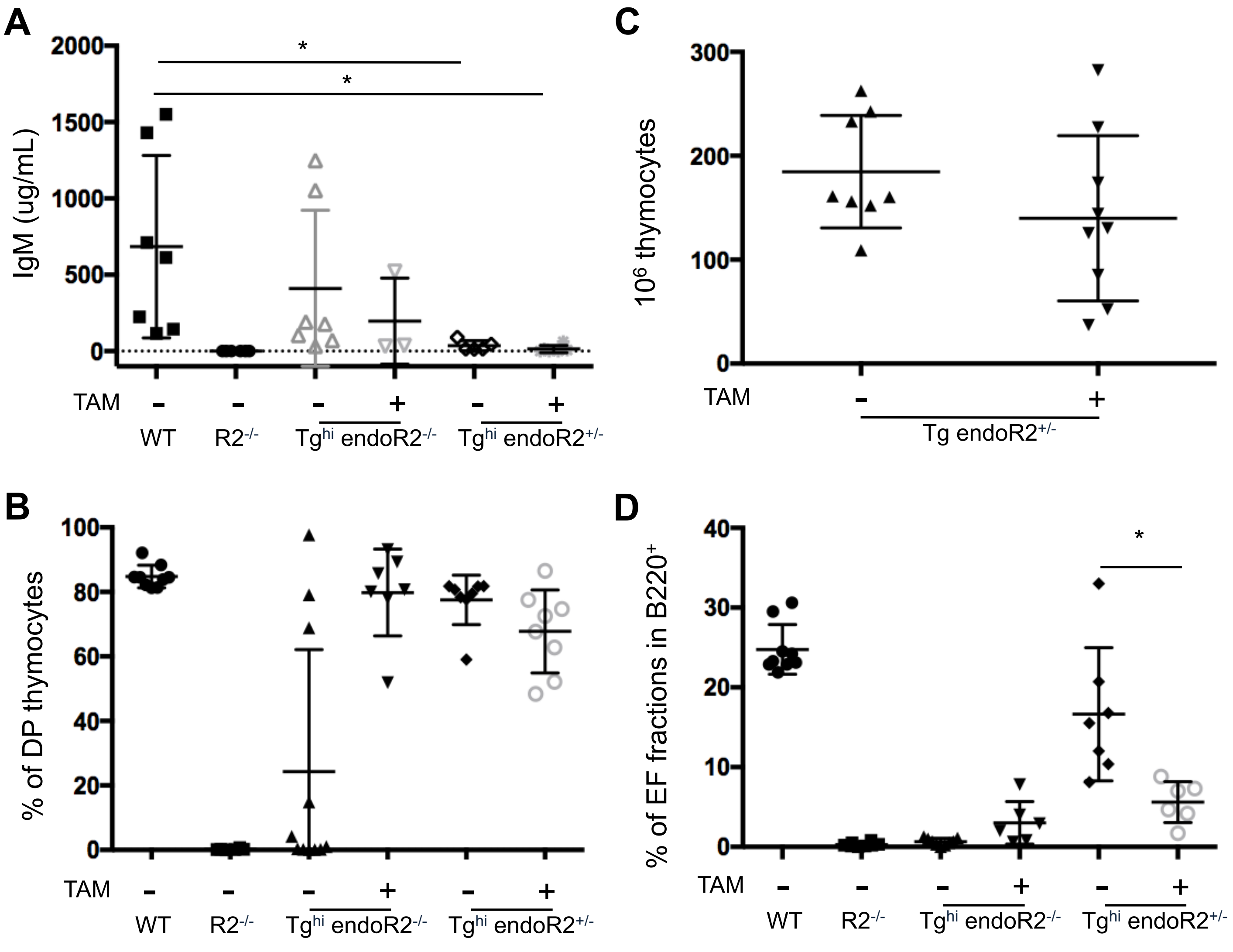

Supplementary Figure 3. **The leacky Tg^{hi} line exhibits defects of lymphoid development.** (**A**) Circulating IgM levels of RAG2-deficient (R2$^{-/-}$), wild type (WT) and Tg^{hi} mice Rag2-incompetent (Tg^{hi} endoR2$^{-/-}$) or -competent (Tg^{hi} endoR2$^{+/-}$) fed with normal (-) versus TAM food (+) are shown. IgM levels in WT mice sera are significantly higher than in Tg^{hi} mice. (* p-val = 0,03 and ** p-val = 0,008 compared to untreated Tg^{hi} endoR2$^{-/-}$ and TAM^{+} Tg^{hi} endoR2$^{+/-}$ mice respectively). WT mice display significantly higher levels of IgM than Tg^{hi} mice (* p-val = 0,04 and p-val = 0,02 compared to untreated and TAM-induced Tg^{hi} endoR2$^{+/-}$ mice respectively). (**B**) Percentages of DP cells in thymocytes show a defect of lymphoid differentiation in TAM^{+} Tg^{hi} endoR2$^{+/-}$ mice. (**C**) The Tg line (low expressor, RAG competent) does not exhibit lower thymocytes numbers upon TAM induction. (**D**) EF fraction cells in B220^{+} bone marrow cells are also diminished upon TAM-induction of Tg^{hi} endoR2$^{+/-}$ mice.

Supplementary Figure 4

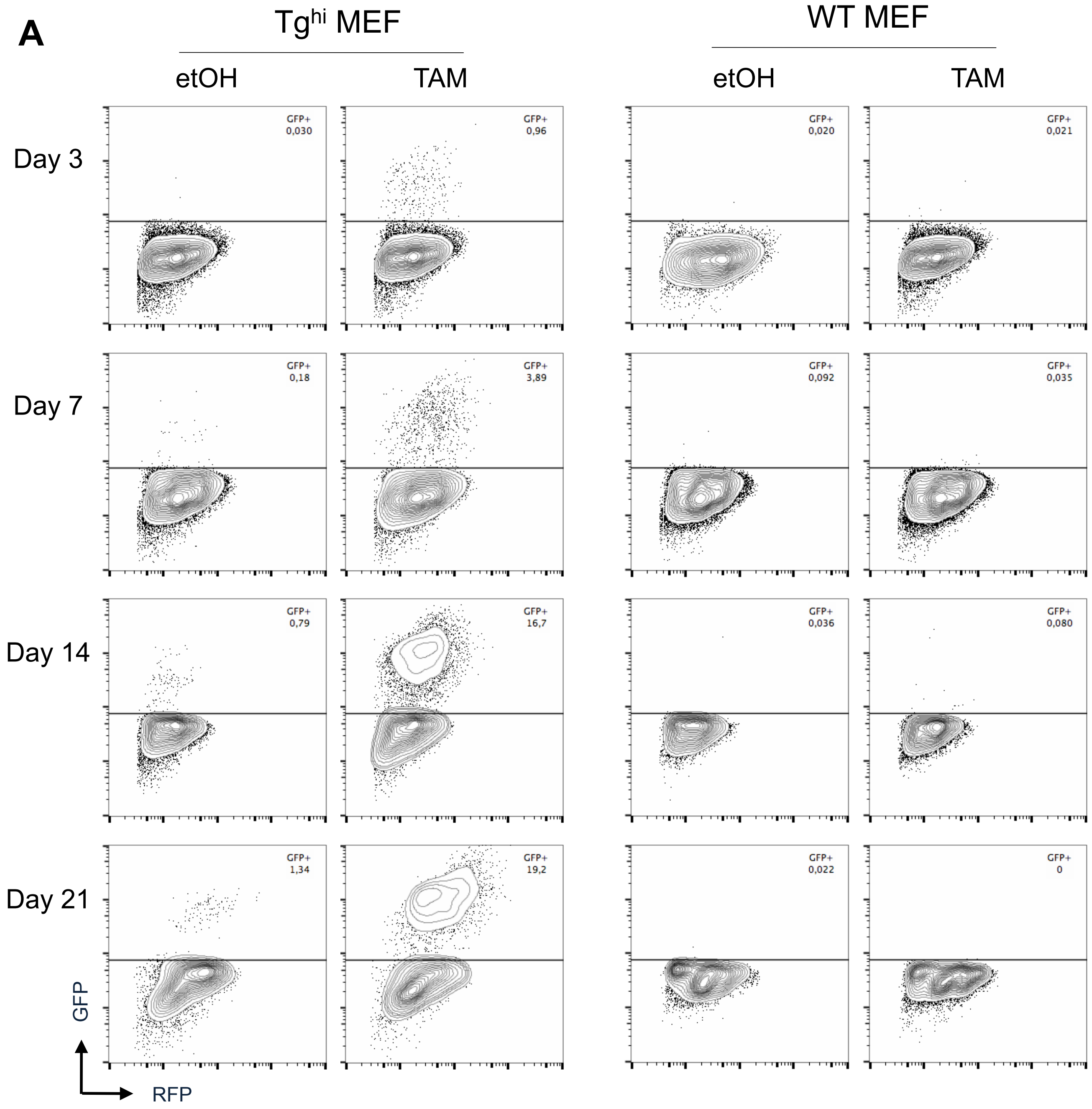

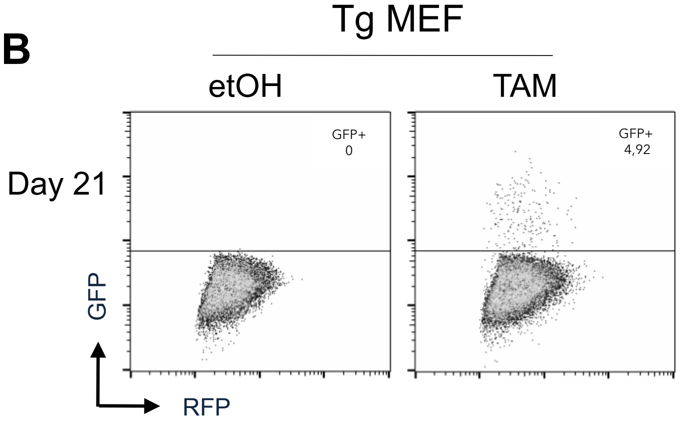

Supplementary Figure 4. **TAM-induction of the *rag*2 transgene in MEF shows RAG activity in non-lymphoid organs.** (**A**) Evolution of FACS profiles of Tg^{hi} and WT MEF treated either by 2% ethanol (etOH) or 200nM of 2% ethanol 4-hydroxy-tamoxifen (TAM), assessed every week. Presence of GFP MEF transgenic MEF treated by TAM only accounts for induced RAG activity from transgenic origin. (**B**) FACS profiles of Tg MEF 4 weeks post induction shows a lower proportion of GFP-expressing cells.

Supplementary Table 1: Sequences of primers used for RT-PC[illegible]

Primer name	Primer Sequence
B-Tubuline FW	GGTGGATCTAGAACCTGGG
B-tubuline RV	CCCAGTGAGTGGGTCAGC
RAG1-endo/tg FW	GAG GTT CCG CTA CGA CTC TG
RAG1-endo/tg RV	TGG CAA TGT GCT AGG TGC TA
RAG1-tg FW	CAA CTC ACA GCG TTT CGC GG
RAG1-tg RV	GAA TTC TTT GCC AAA GTG ATG G
RAG2-endo/tg FW	CCT CTC TAA GAT AAA AGA CC
RAG2-endo/tg RV	TCC CTC GAC TAT ACA CCA CGT CAA
RAG2-tgER FW	TCA ACG GAG CTC AAT AAA CC
RAG2-tgER RV	GCG GTT CAG CAT CCA ACA AG

Supplementary Table 2: RT-PCR conditions

	**Beta-Tubulin**	***rag*1 endo/tg *rag*2 endo/tg**	***rag*1 tg**	***rag*2 tg-ER**
**First denaturation step**	94°C/1min	94°C/ 5min	94°C/5min	94°C/5min
**Amplification step1: number of cycles**	35	4	4	4
**Denaturation**	94°C/1min	94°C/ 5min	94°C/5min	94°C/5min
**Annealing**	58°C/30sec	58°C/ 30sec	60°C/30sec	54°C/30sec
**Elongation**	72°C/30sec	72°C/1,5min	72°C/30sec	72°C/30sec
**Amplification step2: number of cycles**	-	31	28	26
**Denaturation**	-	94°C/ 30sec	94°C/30sec	94°C/30sec
**Annealing**	-	58°C/ 30sec	60°C/30sec	54°C/30sec
**Elongation**	-	72°C/1,5min	72°C/30sec	72°C/30sec
**Last elongation step**	72°C/7min	72°C/ 7min	72°C/7min	72°C/7min

Supplementary Table 3: List of fluorochrome-coupled antibodies used in FACS experiments

Epitope	Clone*
CD4	RM4-5
CD8	YTS169.4
CD44	IM7
CD25	PC61
CD43	S7
B220	RA3-6B2
IgM	R331.24.12
IgD	1.19
Ter	TER-119
TCRb	H57_597
Thy1.2	30H12
CD19	1D3
-CD16/CD32	2.4G2

* All antibodies used were purchased from BD Pharmingen™ or in house produced.
